# Applying a systemic approach that extends beyond the brain to Alzheimer’s disease pathogenesis

**DOI:** 10.1038/s43856-026-01831-z

**Published:** 2026-09-03

**Authors:** Payam Barnaghi, Antigone Fogel, Chloe Walsh, Ramin Nilforooshan, Valeria Ricotti, Thomas Voit

**Affiliations:** 1 Department of Brain Sciences, Imperial College London, London, UK; 2 UK Dementia Research Institute, Care Research and Technology Centre, London, UK; 3 Great Ormond Street Institute of Child Health, University College London, London, UK; 4 Data Research, Innovation and Virtual Environments Unit, Great Ormond Street Hospital, London, UK; 5 NIHR Great Ormond Street Hospital Biomedical Research Centre, University College London, London, UK; 6 Surrey and Borders Partnership NHS Foundation Trust, Leatherhead, UK; 7 Vesalic Limited, London, United Kingdom

**Keywords:** Alzheimer's disease, Alzheimer's disease

## Abstract

Barnaghi et al. propose reframing Alzheimer's disease as a systemic disorder rooted in lysosomal and mitochondrial dysfunction, rather than solely a brain-confined condition. This whole-body perspective explains the links to metabolic, cardiovascular, and inflammatory comorbidities and highlights windows for early intervention.

## Teaser

Traditional dementia research often isolates single aspects, biomarkers or symptoms, overlooking complex interactions shaping disease risk and progression. Lysosomal and mitochondrial dysfunction, reflecting systemic processes, may explain links between Alzheimer’s and metabolic, inflammatory, cardiovascular, ageing, genetic, environmental, and endocrine factors.

Alzheimer’s disease is a progressive neurodegenerative condition characterised by amyloid-*β* and phospho-tau pathology, causing synaptic and neuronal loss that leads to decline in memory, cognition, and ability to perform daily tasks. Considering Alzheimer’s disease as a systemic disorder could transform our understanding of its origins and progression. By adopting a whole-system approach to Alzheimer’s research, we may uncover pathways and mechanistic insights that have long eluded traditional studies, paving the way for integrated strategies that target cellular dysfunction throughout the body. The development and progression of neurodegenerative conditions involve complex interactions among physiological, genetic, metabolic, hormonal, and pathological changes over time. The exact causal mechanisms of neurodegenerative conditions such as Alzheimer’s disease remain unclear, and the pathophysiological changes in the brain and body during the prodromal stage are not well understood^1^. Significant changes in the physiopathology of body systems occur before and after the onset of these conditions. Several studies suggest that Alzheimer’s disease progression involves complex, multi-scale interactions across genetic, metabolic, proteomic, and physiological domains. However, current approaches are limited in their ability to capture and interpret the complex interactions and interconnections among these dynamics^2^.

This systemic framing is supported by recent large-scale plasma proteomic studies of dementia cohorts, which identify circulating protein signatures associated with neurodegeneration and shared across distinct neurodegenerative conditions^3,4^. Such peripheral signatures are difficult to reconcile with a brain-focused model alone, but follow naturally if the underlying pathology reflects a common breakdown in cellular clearance and energy regulation, expressed throughout the body^5^. The fact that these signatures are detectable in blood is significant in itself. It places markers of the proposed systemic dysfunction within reach of routine, longitudinal measurement. This opens the possibility of tracking cellular dysregulation across the lifespan and identifying the critical time points, or windows of opportunity, at which interventions are more likely to alter the disease trajectory. A whole-body, longitudinal view of these markers may therefore reveal mechanistic links that brain-centred, cross-sectional studies often overlook.

The case for a systemic view of neurodegenerative conditions such as Alzheimer’s disease is reinforced by recent therapeutic trials. Anti-amyloid monoclonal antibodies achieve substantial amyloid clearance; however, they yield only modest slowing of clinical decline^6^. If amyloid were the principal driver of disease, effective clearance would be expected to produce a correspondingly significant clinical benefit. This gap between biological target engagement and clinical outcome, together with the limited effect on long-term disease trajectory, instead suggests that amyloid is one component of a broader, multifactorial process, and that effective disease modification may require strategies that address underlying systemic dysfunction rather than targeting a single downstream target.

## Current limitations in Alzheimer’s research:

Previous studies have investigated the links between neurodegeneration, autophagy, and lysosomal and mitochondrial dysfunction, primarily focusing on genetic and molecular evidence. Existing evidence suggests that dysregulation of metabolic and vesicular clearance, the process by which cells regulate their energy and remove waste and damaged components, may be a mechanism underlying neurodegeneration^7^. However, these studies have not fully considered the complex interactions among biophysiological, genetic, and pathological changes, along with their associated comorbidities, in the context of neurodegeneration.

Ageing is a primary risk factor for Alzheimer’s disease. However, several other comorbidities are associated with or considered significant risk factors for the disease^8^. Some of the most well-studied risk factors include cardiovascular disease, diabetes mellitus, and genetic factors such as *APOE*-*ϵ*4 status. Nevertheless, these conditions are often examined separately or only in relation to a limited set of variables, such as metabolism or endocrinology. By approaching Alzheimer’s from a whole-body systems perspective and considering a wide range of conditions and comorbidities that impact individuals throughout their lifespan, researchers can investigate their combined association with increased disease risk. This approach may reveal new mechanistic insights and identify critical periods as “windows of opportunity” for early intervention in high-risk populations.

## Alzheimer’s disease as a systemic disorder:

Alzheimer’s disease progresses gradually over decades. Amyloid and tau pathology begin to accumulate years before the onset of clinical symptoms^9^. The systemic changes associated with the gradual deregulation of cellular clearance and energy metabolism, and their interaction with comorbidities that accrue across the lifespan, plausibly precede and contribute to the earliest amyloid changes in the brain. Considering these systemic changes, the long preclinical phase is not a silent prelude to disease but rather the period during which systemic dysfunction develops, defining an extended window in which it might be detected and the disease trajectory altered. Many of the known risk factors and comorbidities associated with Alzheimer’s disease share an underlying cellular change linked to lysosomal and mitochondrial dysfunction. Typical ageing involves dysregulation of lysosomal and mitochondrial function, and several other factors, including endocrine changes, environmental toxins, inflammation, oxidative stress, cardiovascular conditions, and iatrogenic causes, are also associated with this dysfunction. For example, post-menopausal decline in oestrogen is linked to lysosomal dysregulation, and metabolic stress in conditions such as type 2 diabetes is associated with dysregulation of lysosomal and mitochondrial function^10^. The gut-brain axis offers a further example of this systemic perspective. Gut dysbiosis is associated with chronic systemic inflammation, impaired metabolic regulation, and increased Alzheimer’s risk^11^, and microbial and metabolic signalling from the gut can influence both neuroinflammation and central nervous system function^12^. The gut is another organ system in which disrupted homoeostasis contributes to the cumulative, whole-body effects associated with lysosomal and mitochondrial dysfunction.

Immune dysregulation is another systemic contributor. Ageing- and comorbidity-related cellular changes, including cellular senescence, could drive an inflammatory state that affects both central immune responses to protein aggregates in the brain and peripheral immune function, which in turn influence disease risk and trajectory^13,14^.

**Fig. 1:**
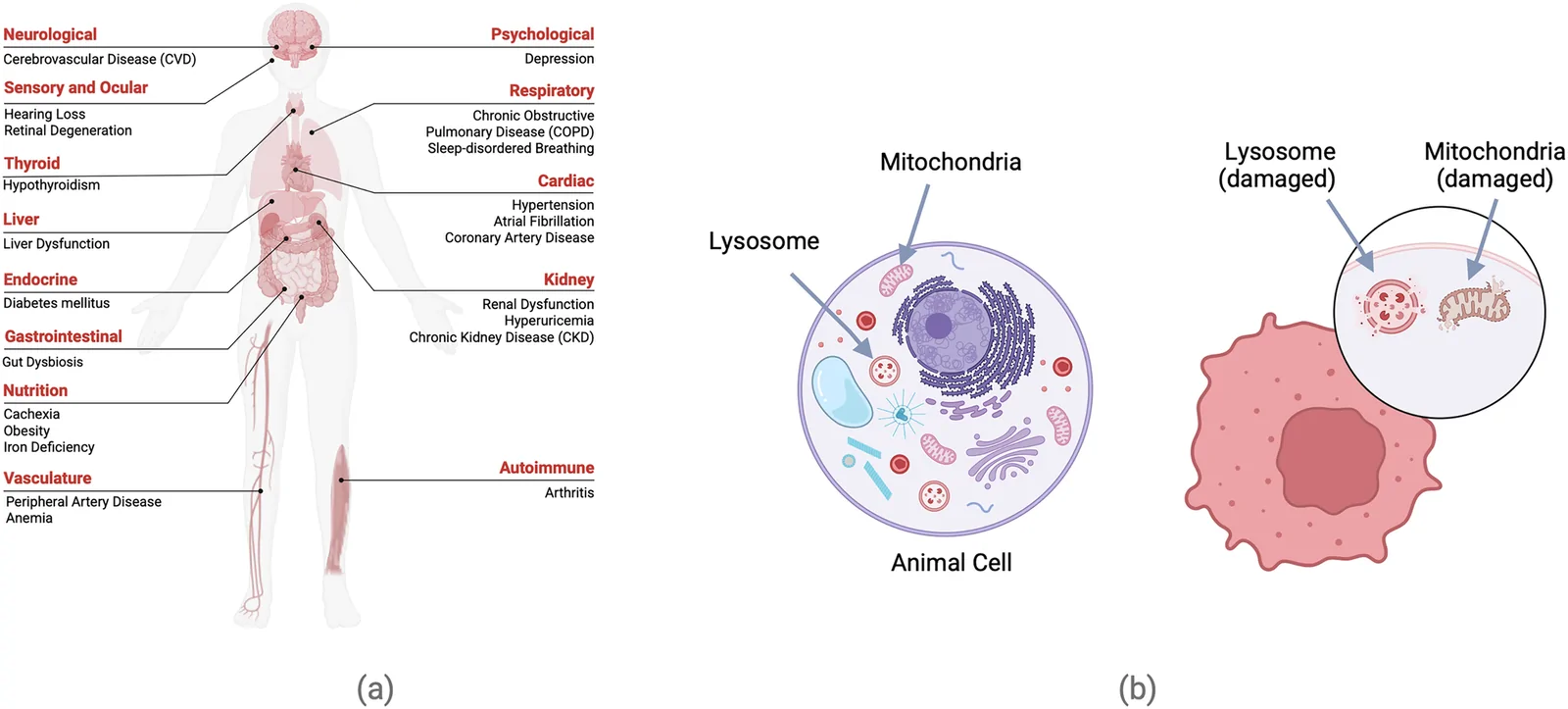
Whole-body perspective illustrating organ-specific comorbidities and their links to lysosomal and mitochondrial dysfunction. **a** Comorbidities and conditions associated with Alzheimer’s that are also associated with systemic breakdown of cellular energy and clearance. **b** Lysosomes and mitochondria within the cell structure; the lysosome and mitochondria membranes become damaged due to various factors. In a compromised unit, the membranes become permeabilised, allowing the flow of molecules and materials that are normally excluded. Created in BioRender https://BioRender.com/deasdoc.

Lysosomes are responsible for recycling and disposing of cellular waste by breaking down waste material, dead cells, and damaged organelles into smaller molecules, thereby regulating the cell’s metabolic system. Mitochondria produce most of the cellular energy in the form of adenosine triphosphate (ATP) through cellular respiration^15^. Since neuronal cells live longer than most other cells in the body, lysosomal and mitochondrial dysfunction have a disproportionately greater impact on metabolic regulation and the clearance of cellular waste in these cells. The aggregation of amyloid and hyperphosphorylated tau, which is linked to mitochondrial and lysosomal dysfunction, further supports this mechanism in Alzheimer’s disease brain pathology. Figure 1 illustrates a whole-body view of organ-related comorbidities described in our previous work^16^ and their association with lysosomal and mitochondrial dysfunction^17,18^.

Considering Alzheimer’s pathogenesis from a systemic perspective, as a manifestation of whole-body cellular dysregulation, provides a unified explanation for the associations between Alzheimer’s disease and conditions related to metabolic, inflammatory, cardiovascular, and endocrine variations. Age, genetics, environmental exposures, comorbidities, and other factors may all contribute cumulatively to an individual’s risk and progression towards Alzheimer’s disease.

A systemic view should also consider protective factors, not only risks. Physical activity acts across the whole body, improving metabolic, cardiovascular, and inflammatory health and reducing the burden of several comorbidities associated with Alzheimer’s disease. Multidomain lifestyle interventions that include physical activity, such as the FINGER^19,20^ and US POINTER^21,22^ studies, slow cognitive decline in at-risk older adults.

Considering the translation of this framework into practice points to three directions. First, longitudinal, multi-organ data linking electronic health records, peripheral biomarkers, and remote digital and wearable monitoring would enable tracking of systemic dysfunction across the lifespan and testing it as a precursor to, rather than a consequence of, neurodegeneration. Second, the convergence of comorbidities with lysosomal and mitochondrial dysfunction identifies measurable readouts of cellular clearance and energy metabolism as candidate early markers and targets for therapies that act on these shared pathways rather than on a single downstream target, such as amyloid-*β* or tau. Third, treating accumulated comorbidity^16^ as a modifiable contributor to risk reframes the management of metabolic, cardiovascular, and inflammatory conditions in midlife as a potential route to delaying or preventing neurodegeneration^23^ and defines the windows of opportunity in which such intervention is more likely to alter the disease trajectory^24^.

The pathology and progression of Alzheimer’s disease extend beyond the brain; it is a systemic breakdown of cellular clearance and energy regulation, a perspective that requires a paradigm shift in our approach to studying and attempting to treat this condition. A systemic model also raises the question of disease specificity. The contributors discussed, such as ageing, metabolic and cardiovascular comorbidities, inflammation, and impaired cellular clearance and energy metabolism, are common across neurodegenerative diseases, with, for example, Alzheimer’s and Parkinson’s sharing several risk factors and comorbidities^25,26^. We suggest that systemic dysfunction acts as a common foundation, while the clinical phenotype is influenced by regional, cell-type, and network-specific vulnerabilities^27,28^. These interact with genetic predispositions and the specific proteins that aggregate in each disease. Thus, the key difference between Alzheimer’s and other neurodegenerative conditions lies less in systemic dysfunction itself and more in where and in which cell populations this dysfunction first exceeds the degeneration threshold. Understanding the factors behind this selective vulnerability remains a fundamental open question, and a systemic, whole-body approach would be well-suited to investigating it.

## Supplementary information

Supplementary information
