# Supplementary material for: Applying a systemic approach that extends beyond the brain to Alzheimer’s disease pathogenesis
Source: Commun Med (Lond). 2026 Sep 3;6:475. doi: 10.1038/s43856-026-01831-z (PMC13542206; doi:10.1038/s43856-026-01831-z)
Supplement: Supplementary file 1 [file 43856_2026_1831_MOESM1_ESM.pdf]

nature portfolio

Peer Review File

Applying a systemic approach that extends beyond the brain to Alzheimer’s disease pathogenesis

Corresponding Author: Professor Payam Barnaghi

This file contains all reviewer reports in order by version, followed by all author rebuttals in order by version.

Version 0:

Reviewer comments:

Reviewer #1

(Remarks to the Author)
In this brief commentary, Barnaghi et al. advocate for a systemic, holistic perspective on Alzheimer's disease (AD). Building on their previous work, the authors propose that lysosomal and mitochondrial dysfunction — linked to whole-body cellular dysregulation — provides a unified explanation for the well-documented associations between AD and comorbidities prevalent in the aging population (including type 2 diabetes, cardiovascular disease, and chronic inflammation). While the proposed framework is not entirely novel, it has never been comprehensively or effectively pursued (see, for instance, PMID: 24747741), and the authors' synthesis deserves credit for its integrative scope.
The core message of this commentary would be substantially strengthened by engagement with recent large-scale proteomic studies conducted on blood samples from dementia patients (PMID: 40665052; PMID: 40665048). These studies provide empirical support for the systemic dysregulation hypothesis and, importantly, point to shared, trans-diagnostic proteomic signatures across neurodegenerative conditions — a finding directly relevant to the authors' argument. Additionally, the recent, largely modest results from single-target interventions in AD — most notably anti-amyloid monoclonal antibodies — further underscore the multifactorial and systemic nature of the disease (see PMID: 38065226). A brief discussion of this translational evidence would considerably reinforce the authors' call for a paradigm shift.
The paper centers heavily on lysosomes and mitochondria but says very little about how this systemic framework should translate into actionable research or clinical settings. The authors would strengthen their case by sketching even a minimal roadmap or mentioning active research strategies focusing on these targets.

Minor points:
The gut–brain axis is absent from the systemic map, despite substantial evidence linking gut dysbiosis to neuroinflammation and AD risk.
Dysregulation should be used instead of deregulation.

Reviewer #2

(Remarks to the Author)
Barnaghi and colleagues present an argument for viewing Alzheimer’s disease as a systemic disease that is likely the culmination of dysfunction in lysosomes (to clear cellular waste) and mitchochondria (to produce cellular energy). They propose that such long-term changes, in combination with ageing, genetics, metabolic disorders, cardiovascular comorbidities and environmental factors contribute to one’s overall risk of developing Alzheimer’s disease. The perspective article urges broadening our view of contributions by these systemic factors to better understand Alzheimer’s disease both before and during its progress, and to find novel strategies to prevent and treat the disease. Overall, it is an interesting perspective that has been gaining credence through new data and conference discussions.

Comments to consider:
1. Alzheimer’s is a very slow, chronic disease. Perhaps the authors could mention that these long “festering” changes precede or begin at the start of amyloid changes in the brain and last for years.
2. Immune dysregulation is also thought to contribute to Alzheimer’s disease. While cellular senescence may play a role in the ability of immune cells to response successfully to the buildup of proteins in the brain, it may also affect system immune responses in the periphery that might affect the brain. Some mention of immune dysregulation might be helpful here.
3. Physical activity affects the whole body and is thought to protect against many of the comorbidities mentioned in this

article. Physical activity also reduces the age of onset of Alzheimer’s disease according to the FINGERS and US POINTER lifestyle intervention studies. Perhaps this is a good example of a systemic beneficial effect that delays onset of Alzheimer’s disease.
4. How much of these systemic contributions are specific for Alzheimer’s disease vs. neurodegenerative diseases in general? What are the factors that set one person up for Alzheimer’s disease and another up for Parkinson’s disease (or other neurodegenerative diseases)?
5. Lastly, while this is a topic of increasing interest in the Alzheimer’s disease field, this short perspective seems to repeat many phrases multiple times and does not give a lot of direct examples. It would be improved by including more examples and providing a bit more detail. For example, inflammatory pathways are barely touched upon and yet inflammaging is important in neurodegenerative diseases.

Version 1:

Reviewer comments:

Reviewer #1

(Remarks to the Author)
The authors adequately addressed the concerns I raised in the first round of revision.

Reviewer #2

(Remarks to the Author)
The authors have done an outstanding job responding to the reviewers' comments. They have incorporated more explicit details and examples (with references) to support their perspective. The manuscript is now easier to follow and will inspire future studies into AD and likely, other neurodegenerative diseases. Well done!

Open Access This Peer Review File is licensed under a Creative Commons Attribution 4.0 International License, which permits use, sharing, adaptation, distribution and reproduction in any medium or format, as long as you give appropriate credit to the original author(s) and the source, provide a link to the Creative Commons license, and indicate if changes were made.
In cases where reviewers are anonymous, credit should be given to 'Anonymous Referee' and the source.
The images or other third party material in this Peer Review File are included in the article’s Creative Commons license, unless indicated otherwise in a credit line to the material. If material is not included in the article’s Creative Commons license and your intended use is not permitted by statutory regulation or exceeds the permitted use, you will need to obtain permission directly from the copyright holder.
To view a copy of this license, visit https://creativecommons.org/licenses/by/4.0/

Dear Editorial team and the reviewers,

We thank both reviewers for their careful and constructive assessment of our manuscript, and for recognising the value of a systemic perspective on Alzheimer's disease. The comments have helped us sharpen the argument and ground it in more concrete evidence and examples.

We have revised the manuscript to address every point raised. In doing so, we have included brief descriptions and citations to recent large-scale plasma proteomic studies and their trans-diagnostic signatures, added the translational evidence from anti-amyloid trials and from multidomain lifestyle interventions, extended the systemic map to include the gut-brain axis and immune dysregulation, and sketched a concrete roadmap for translating the framework into research and clinical strategies. We have also clarified the question of disease specificity across neurodegenerative conditions and tightened the prose throughout to reduce repetition and add worked examples.

We are grateful for the feedback and suggestions that have helped strengthen the manuscript, and we believe the revised perspective is clearer, more concrete, and better supported as a result. Our point-by-point responses follow below; reviewer comments are shown in italics, with our responses below each and the corresponding changes shown.

Reviewers' comments:

Reviewer #1 (Remarks to the Author):

In this brief commentary, Barnaghi et al. advocate for a systemic, holistic perspective on Alzheimer's disease (AD). Building on their previous work, the authors propose that lysosomal and mitochondrial dysfunction — linked to whole-body cellular dysregulation — provides a unified explanation for the well-documented associations between AD and comorbidities prevalent in the aging population (including type 2 diabetes, cardiovascular disease, and chronic inflammation). While the proposed framework is not entirely novel, it has never been comprehensively or effectively pursued (see, for instance, PMID: 24747741), and the authors' synthesis deserves credit for its integrative scope.

R.1.1*: The core message of this commentary would be substantially strengthened by engagement with recent large-scale proteomic studies conducted on blood samples from dementia patients (PMID: 40665052; PMID: 40665048). These studies provide empirical support for the systemic dysregulation hypothesis and, importantly, point to shared, trans-diagnostic proteomic signatures across neurodegenerative conditions — a finding directly relevant to the authors' argument.*

We would like to thank the reviewer for the careful and constructive reading of our commentary, and for recognising the integrative scope of the proposed framework. We also appreciate the reviewer pointing us to PMID: 24747741, which we agree provides an important precedent; we now cite this work when noting that a systemic framing has been proposed before but not pursued comprehensively.

Regarding the references to recent large-scale plasma proteomic studies (PMID: 40665052; PMID: 40665048)**:** We agree; these are valuable additions. These studies strengthen the central argument of the commentary by providing references to large-scale support for systemic dysregulation in dementia, measured directly in peripheral blood. Their identification of shared, trans-diagnostic proteomic signatures across neurodegenerative conditions aligns closely with our proposal that lysosomal and mitochondrial dysfunction represents a common, whole-body axis rather than a brain-confined process.

We have revised the manuscript to include references to these (lines 30-38).

"This systemic framing is supported by recent large-scale plasma proteomic studies of dementia cohorts, which identify circulating protein signatures associated with neurodegeneration and shared across distinct neurodegenerative conditions~\cite{Imam-2025, Ali-2025}. Such peripheral

signatures are difficult to reconcile with a brain-focused model alone, but follow naturally if the underlying pathology reflects a common breakdown in cellular clearance and energy regulation, expressed throughout the body~\cite{Morris-2014}."

R.1.2. *Additionally, the recent, largely modest results from single-target interventions in AD — most notably anti-amyloid monoclonal antibodies — further underscore the multifactorial and systemic nature of the disease (see PMID: 38065226). A brief discussion of this translational evidence would considerably reinforce the authors' call for a paradigm shift.*

We agree, and this strengthens the argument considerably. Thank you for the suggestion. The modest clinical effect sizes of anti-amyloid monoclonal antibodies, despite clear target engagement and amyloid clearance, are difficult to reconcile with a model in which amyloid is the sole or principal driver of disease. This is precisely the translational evidence on which our commentary's call for a paradigm shift rests, and we are grateful to the reviewer for suggesting further clarification on this.

We have revised the manuscript to include references to this (lines 40-46).

"The case for a systemic view of neurodegenerative conditions such as Alzheimer's disease is reinforced by recent therapeutic trials. Anti-amyloid monoclonal antibodies achieve substantial amyloid clearance; however, they yield only modest slowing of clinical decline~\cite{Granzotto-2024}. If amyloid were the principal driver of disease, effective clearance would be expected to produce a correspondingly significant clinical benefit. This gap between biological target engagement and clinical outcome, together with the limited effect on long-term disease trajectory, instead suggests that amyloid is one component of a broader, multifactorial process, and that effective disease modification may require strategies that address underlying systemic dysfunction rather than targeting a single downstream target."

R1.3*. The paper centers heavily on lysosomes and mitochondria but says very little about how this systemic framework should translate into actionable research or clinical settings. The authors would strengthen their case by sketching even a minimal roadmap or mentioning active research strategies focusing on these targets.*

Thank you for this excellent suggestion. We have added a short closing paragraph that sketches a brief roadmap across three concrete directions, without overstating what a commentary can deliver.

We have revised the manuscript to include this (lines 102-110).

"Considering the translation of this framework into practice points to three directions. First, longitudinal, multi-organ data linking electronic health records, peripheral biomarkers, and remote digital and wearable monitoring would enable tracking of systemic dysfunction across the lifespan and testing it as a precursor to, rather than a consequence of, neurodegeneration. Second, the convergence of comorbidities with lysosomal and mitochondrial dysfunction identifies measurable readouts of cellular clearance and energy metabolism as candidate early markers and targets for therapies that act on these shared pathways rather than on a single downstream target, such as amyloid-β or tau. Third, treating accumulated comorbidity~\cite{Walsh-2025} as a modifiable contributor to risk reframes the management of metabolic, cardiovascular, and inflammatory conditions in midlife as a potential route to delaying or preventing neurodegeneration~\cite{Merten-2023} and defines the windows of opportunity in which such intervention is more likely to alter the disease trajectory~\cite{Biggart-2025}."

Minor points:
R.1.4. *The gut–brain axis is absent from the systemic map, despite substantial evidence linking gut dysbiosis to neuroinflammation and AD risk.*

We thank the reviewer for identifying this omission. The gut–brain axis is well supported and fits within the systemic framework; its absence from the map was an oversight. We have addressed this in two places.

We have added the gastrointestinal system to Figure 1(a), listing gut dysbiosis under a new "Gastrointestinal" label. We also added the following to the *A Systemic Perspective* section (lines 74-77).

“The gut-brain axis offers a further example of this systemic perspective. Gut dysbiosis is associated with chronic systemic inflammation, impaired metabolic regulation, and increased Alzheimer's risk~\cite{Kumari-2025}, and microbial and metabolic signalling from the gut can influence both neuroinflammation and central nervous system function~\cite{Zhu-2017}. The gut is another organ system in which disrupted homeostasis contributes to the cumulative, whole-body effects associated with lysosomal and mitochondrial dysfunction.”

R.1.5. *Dysregulation should be used instead of deregulation.*

Thank you for noticing this. We have fixed this in the text.

Reviewer #2 (Remarks to the Author):

Barnaghi and colleagues present an argument for viewing Alzheimer’s disease as a systemic disease that is likely the culmination of dysfunction in lysosomes (to clear cellular waste) and mitochochondria (to produce cellular energy). They propose that such long-term changes, in combination with ageing, genetics, metabolic disorders, cardiovascular comorbidities and environmental factors contribute to one’s overall risk of developing Alzheimer’s disease. The perspective article urges broadening our view of contributions by these systemic factors to better understand Alzheimer’s disease both before and during its progress, and to find novel strategies to prevent and treat the disease. Overall, it is an interesting perspective that has been gaining credence through new data and conference discussions.

Comments to consider:

R.2.1. *Alzheimer’s is a very slow, chronic disease. Perhaps the authors could mention that these long “festering” changes precede or begin at the start of amyloid changes in the brain and last for years.*

We thank the reviewer for highlighting this, which strengthens the temporal dimension of our argument. Alzheimer's disease has an extended preclinical phase, and the systemic changes we describe plausibly begin before, and accumulate alongside, the earliest amyloid pathology. We have clarified this further.

We have revised the manuscript to include this (lines 63-68).

“Alzheimer's disease develops over decades. Amyloid and tau pathology begin to accumulate years before the onset of clinical symptoms~\cite{Insel-2021}. The systemic changes associated with the gradual deregulation of cellular clearance and energy metabolism, and their interaction with comorbidities that accrue across the lifespan, plausibly precede and contribute to the earliest amyloid changes in the brain. Considering these systemic changes, the long preclinical phase is not a silent prelude to disease but rather the period during which systemic dysfunction develops, defining an extended window in which it might be detected and the disease trajectory altered.”

R.2.2. *Immune dysregulation is also thought to contribute to Alzheimer’s disease. While cellular senescence may play a role in the ability of immune cells to response successfully to the buildup of*

proteins in the brain, it may also affect system immune responses in the periphery that might affect the brain. Some mention of immune dysregulation might be helpful here.

We agree, and this adds an important dimension. Immune dysregulation is a strong candidate mechanism linking systemic and central processes, and cellular senescence provides a plausible bridge between the two. We have added the following (lines 79-81):

"Immune dysregulation is another systemic contributor. Ageing- and comorbidity-related cellular changes, including cellular senescence, could drive an inflammatory state that affects both central immune responses to protein aggregates in the brain and peripheral immune function, which in turn influence disease risk and trajectory~\cite{Gol-2025, Cao-2025}."

R.2.3. *Physical activity affects the whole body and is thought to protect against many of the comorbidities mentioned in this article. Physical activity also reduces the age of onset of Alzheimer's disease according to the FINGERS and US POINTER lifestyle intervention studies. Perhaps this is a good example of a systemic beneficial effect that delays onset of Alzheimer's disease.*

We thank the reviewer for this suggestion. Physical activity is a strong example of a systemic intervention with a systemic benefit, and it complements our argument well. We have added the following paragraph (lines 97-100).

"A systemic view should also consider protective factors, not only risks. Physical activity acts across the whole body, improving metabolic, cardiovascular, and inflammatory health and reducing the burden of several comorbidities associated with Alzheimer's disease. Multidomain lifestyle interventions that include physical activity, such as the FINGER ~\cite{ Kivipelto-2013, Ngandu-2015} and US POINTER~\cite{Baker-2024, Espeland-2025 } studies, slow cognitive decline in at-risk older adults."

R.2.4. *How much of these systemic contributions are specific for Alzheimer's disease vs. neurodegenerative diseases in general? What are the factors that set one person up for Alzheimer's disease and another up for Parkinson's disease (or other neurodegenerative diseases)?*

This is a very important question, and we do not think it has a complete answer yet. We have addressed it directly in the manuscript rather than implying more specificity than the evidence supports. Our view is that the systemic contributors we describe are largely shared across neurodegenerative conditions, which is also consistent with the trans-diagnostic proteomic signatures noted above (R.1.1) and with the substantial comorbidity and risk-factor overlap between conditions such as Alzheimer's and Parkinson's disease. What appears to differ is not the systemic substrate itself, but where its consequences are first and most severely expressed. We have added the following to the closing (lines 113-122):

"A systemic model also raises the question of disease specificity. The contributors discussed, such as ageing, metabolic and cardiovascular comorbidities, inflammation, and impaired cellular clearance and energy metabolism, are common across neurodegenerative diseases, with, for example, Alzheimer's and Parkinson's sharing several risk factors and comorbidities~\cite{ Rosen-2007, Chen-2026}. We suggest that systemic dysfunction acts as a common foundation, while the clinical phenotype is influenced by regional, cell-type, and network-specific vulnerabilities~\cite{Mrdjen-2019, Mrdjen-2019}. These interact with genetic predispositions and the specific proteins that aggregate in each disease. Thus, the key difference between Alzheimer's and other neurodegenerative conditions lies less in systemic dysfunction itself and more in where and in which cell populations this dysfunction first exceeds the degeneration threshold. Understanding the factors behind this selective vulnerability remains a fundamental open question, and a systemic, whole-body approach would be well-suited to investigating it."

R.2.5. *Lastly, while this is a topic of increasing interest in the Alzheimer's disease field, this short*

perspective seems to repeat many phrases multiple times and does not give a lot of direct examples. It would be improved by including more examples and providing a bit more detail. For example, inflammatory pathways are barely touched upon and yet inflammaging is important in neurodegenerative diseases.

We thank the reviewer for this observation. In revising the manuscript to address the comments above, we have substantially increased the number of examples and reduced repetition.

The revisions now provide specific, examples throughout: the gut–brain axis and gut dysbiosis (R.1.4); cellular senescence and immune dysregulation acting on both central and peripheral immune function (R.2.2); physical activity and the multidomain FINGER and US POINTER lifestyle interventions as a systemic protective effect (R.2.3); the modest clinical effects of anti-amyloid monoclonal antibodies as translational evidence for a multifactorial process (R.1.2); and selective vulnerability as the basis for disease specificity across neurodegenerative conditions (R.2.4). We have also added engagement with recent large-scale plasma proteomic studies and their trans-diagnostic signatures (R.1.1).

Specifically, regarding inflammatory pathways, the revised text now addresses inflammation and inflammaging directly through additions on cellular senescence and immune dysregulation (R.2.2), in which chronic inflammation arising from ageing- and comorbidity-related cellular changes is linked to both central and peripheral immune function and to disease risk and trajectory.

In addressing these points, we have revised the manuscript to address this comment. We believe the revised perspective is more concrete and more clearly argued as a result.
